# Panning for gold: Comparative analysis of cross-platform approaches for automated detection of political content in textual data

**DOI:** 10.1371/journal.pone.0312865

**Published:** 2024-11-18

**Authors:** Mykola Makhortykh, Ernesto de León, Aleksandra Urman, Teresa Gil-Lopez, Clara Christner, Maryna Sydorova, Silke Adam, Michaela Maier

**Affiliations:** 1 Institute of Communication and Media Studies, University of Bern, Bern, Switzerland; 2 Social Computing Group, University of Zurich, Zurich, Switzerland; 3 Department of Communication, University Carlos III of Madrid, Madrid, Spain; 4 Institute for Communication Psychology and Media Education, Rheinland-Pfälzische Technische Universität Kaiserslautern-Landau, Landau, Germany; Centro de Investigacion en Ciencias de Informacion Geoespacial AC (Research Center on Geospatial Information Sciences), MEXICO

## Abstract

To understand and measure political information consumption in the high-choice media environment, we need new methods to trace individual interactions with online content and novel techniques to analyse and detect politics-related information. In this paper, we report the results of a comparative analysis of the performance of automated content analysis techniques for detecting political content in the German language across different platforms. Using three validation datasets, we compare the performance of three groups of detection techniques relying on dictionaries, classic supervised machine learning, and deep learning. We also examine the impact of different modes of data preprocessing on the low-cost implementations of these techniques using a large set (n = 66) of models. Our results show the limited impact of preprocessing on model performance, with the best results for less noisy data being achieved by deep learning- and classic machine learning-based models, in contrast to the more robust performance of dictionary-based models on noisy data.

## Introduction

The emergence of the present high-choice media environment [1] has been accompanied by an unprecedented expansion of the volume of politics-related content and the formats in which it is consumed (e.g. microblogs and online newspapers [2]). To understand political information consumption in such an environment as well as measure exposure to political information [3], we need new approaches to trace individual interactions with content online (e.g. clickstream or web-tracking data [4, 5]), but also novel techniques to analyse the content (e.g. to detect the presence of politics-related information [6]).

To date, most studies have relied on the information source (e.g. web domain type) to distinguish the information individuals engage with online [7, 8]. While some studies have also looked at actual content, the usual focus is on a single type of (usually, Anglophone) data, such as journalistic articles [9] or tweets [10]. While such a specific focus is common in natural language processing (NLP) research, to which the “no free lunch” theorem (i.e. the idea that more universal computational approaches will always underperform compared with more specific/narrow ones [11]) is applicable, it limits the possibilities for studying engagements with (political) content that occur across multiple platforms.

In this article, we report results from a comparative analysis of approaches for the automated detection of politics-related information in multi-platform German textual content. Specifically, we compare the performance of three groups of detection techniques based on dictionaries, classic supervised machine learning (CSML), and deep learning (DL) across three validation datasets with varying degrees of text “noise” [12]. In doing so, we also discuss how the performance of the low-cost implementations of these techniques is affected by different modes of text preprocessing. We argue that despite the multiple challenges associated with developing cross-platform content analysis approaches, pursuing this task is important for realising the opportunities enabled by new forms of cross-platform passive data collection [13] and advancing research on diverse communication phenomena, ranging from selective exposure [14] to media effects [15] to news consumption inequalities [16]. Specifically, we suggest that the results of the study will be useful for the research community by helping to identify the advantages and disadvantages of the computational approaches, which are commonly used in the field of (political) communication research.

The rest of the paper is organised as follows. First, we briefly review the existing research on the automated detection of political content and the impact of text preprocessing on automated content analysis techniques. We then introduce the methodology used to compare the performance of different detection models and different modes of preprocessing. After this, we share our findings about the models’ performances across the three validation datasets and discuss the implications of the lessons learned through this process, together with the study’s limitations and directions for future research.

### Automated detection of politics-related information

While the ability to detect politics-related information is important for studying communication in online environments, the practical realisation of this task is complicated by the different formats in which such information can appear. Existing studies often address this challenge by assuming that all content coming from a specific source (e.g. a news website [7]) or of a specific type (e.g. political manifestos [17]) is related to politics. However, the rise of multi-purpose online platforms (e.g. social media [10]), where individuals can engage with content dealing with a broad range of topics (both related and not related to politics), and the new formats through which politics-related information can be disseminated, prompt the need for approaches looking at the presence of such information on the content level. Most of these approaches rely on one of three groups of NLP techniques: dictionary-, CSML-, and DL-based approaches. It is important to note that this is not an exhaustive selection of approaches. There are also, for instance, unsupervised approaches for detecting politics-related information [18]; however, we follow the assumption from [19] that dictionary- and CSML-based approaches remain the most commonly used for complex NLP tasks in the context of political communication.

Dictionary-based techniques utilise lists of terms related to the specific construct to be detected [20]. Compared with more complex CSML- and DL-based techniques, the main principle of dictionary-based approaches is rather simple: if the piece of content contains a certain number of terms present in the dictionary, then it is classified as political. The simplicity and transparency of dictionaries have contributed to their active use for detecting political content based on the presence of certain features like politicians’ or parties’ names [19, 21] or terms associated with a specific politics-related phenomenon (e.g. migration [22]). Dictionaries have also been used to detect political information within content coming from different platforms, such as news websites [23] and social media [21].

CSML-based techniques rely on the likelihood of individual terms being representative of a specific content category [23]. Using manually annotated corpora (e.g. of sentences or documents), these techniques employ statistical models [24] to predict whether a specific piece of content has certain features (e.g. those related to politics). Despite being less transparent than dictionaries, CSML-based techniques involve less preparatory work: instead of a list of terms representative of a particular issue, they require just a set of labelled data (e.g. based on manual annotation or metadata [25, 26]). Together with their higher performance compared with dictionaries as evidenced by comparisons of the performance of these different techniques for other common content analysis tasks (e.g. sentiment detection [27]), their ease of use has contributed to the growing application of CSML-based approaches for politics-related information detection (e.g. on social media [10]).

Compared with CSML, *DL-based techniques* make better use of contextual information and are more capable of processing unstructured data [28]. In particular, convolutional neural networks (CNN) and long short-term memory networks (LSTM) have shown promising performance in text classification [29]. The effectiveness of DL in recognising sequential patterns is amplified by transformer models, such as bidirectional encoder representations from transformers (BERT), which rely on a substantial volume of contextual information [30]. Although notorious for their lack of transparency [31], recent studies have demonstrated the substantial advantages of DL-based approaches in terms of performance on political detection tasks for social media [32] and journalistic content [33].

Despite relying on different techniques to detect politics-related information, the majority of studies noted above share a common feature: the tendency to focus on content coming from a single platform (e.g. Twitter [32]). Such a monoplatform focus limits the applicability of existing approaches to large datasets dealing with cross-platform engagement with online content. The growing availability of such datasets, which are provided, for instance, by web-tracking studies, stresses the importance of more cross-platform approaches for detecting politics-related information. An example of the volume of data used by these studies is given by [26], who worked with 150 million visits from thousands of different web domains. Another study [6] relied on 36.8 million visits, with a median number of different domains visited per user of more than 800.

### Preprocessing and automated content analysis

Preprocessing decreases the amount of noise in textual data by reducing the complexity of textual features [34], which is particularly important for dealing with multi-platform data, where the amount of noise is higher compared with monoplatform data. Some basic modes of preprocessing include letter lowercasing, punctuation removal, and the exclusion of repeating characters [35]. More complex modes deal with stopword removal (i.e. the removal of very common words or those with little meaning, such as “the”); stemming, where words are stripped to their base by removing verb and adverb suffixes (e.g. “ed” and “ly”); and lemmatisation, where inflicted versions of words are converted to their neutral lemma (e.g. “am” and “is” become “be”).

Despite the increasing number of studies conducting systematic analyses of the effects of preprocessing on the performance of automated content analysis approaches [35, 36], the choice of an optimal mode of preprocessing remains a challenging task. Its complexity can be attributed to several factors. First, the majority of studies compare the impact of preprocessing within a specific group of analytical techniques (e.g. CSML [35]) and rarely examine the variation in the effects of preprocessing between different groups of techniques (e.g. CSML and DL). Second, while the effects of preprocessing are influenced by the contextual factors associated with the task (e.g. the language of the dataset), most research focuses on Anglophone textual data, thus limiting the possibilities for investigating this impact. Third, there can be multiple implementations of the same preprocessing mode; while the impact of such differences can be marginal (e.g. different stemmers resulting in less than a 0.01 difference in accuracy scores [37]), it nevertheless causes variation in the technique’s performance.

Under these circumstances, many studies argue for the use of less complex modes of preprocessing that require fewer computational resources but improve model performance. For instance, [35] compared the impact of simpler forms of preprocessing on the performance of three CSML models for Anglophone data and found that stopword removal resulted in the most consistent performance improvement for two out of three of the models. Similarly, the beneficial effect of stopword removal was observed in a study examining the effects of preprocessing on CSML-based techniques for Czech data [38]. However, in some cases, such as when dealing with content in Hebrew [39] or corpora with few stopwords (e.g. spam emails [40]), the removal of stopwords actually worsened the model performance.

Compared with simple modes of preprocessing, such as stopword removal or the reduction of repeated characters, more complex modes (e.g. lemmatisation) enable more feature reduction and thus can provide larger performance increases for techniques affected by data noise (e.g. CSML-based ones [36]). In practice, however, the effect of complex modes of preprocessing turns out to be rather ambiguous: in some use cases, particularly those dealing with CSML, stemming [41], or lemmatisation with stopword removal [42] can result in performance improvements. In other cases, these modes of preprocessing result in marginal improvement [43] or an actual drop in performance [38].

This ambiguous effect of more complex forms of preprocessing is particularly pronounced in the case of DL-based techniques, which sometimes benefit from higher noise (e.g. in the form of stopwords) that enables more possibilities to understand contextual relationships within the corpus. [44] showed that in the case of disaster-related Twitter content in English, stemming and stopword removal led to a decrease in the performance of the BERT because of the elimination of text features. At the same time, [45] found that for Russian language classification tasks using BERT, lemmatisation enabled performance improvement, whereas stemming did not.

## Materials and methods

### Political content detection

#### Defining political content

We understand political content as materials mentioning processes and political procedures (politics), form, structures, and institutional aspects (polity), and the content of political disputes (policy) [46] (for more information on this definition, see Appendix A1 in S1 File). Political content encompasses a wide range of topics, including but not limited to discussions about government policies, electoral processes, political parties, and legislative debates. It also includes commentary on public administration, international relations, and the activities of non-governmental organisations that influence or react to political developments. Furthermore, political content can address social issues such as healthcare, education, and civil rights, which are often subjects of political action and discourse.

Because of our use case (see Appendix A2 in S1 File), we were particularly interested in Swiss/German actors, as well as the political issues relevant to these two countries. The focus on Swiss and German political contexts allowed us to tailor our detection models to specific linguistic and cultural nuances, making our analysis more accurate and contextually relevant. For instance, Swiss political content might frequently mention the Federal Council or referendums, while German political content might often discuss the Bundestag or state elections.

Although this specificity has implications for the direct reuse of the detection models we developed, we expect that the observations generated through our model comparison will be applicable to a broad range of contexts. The underlying principles and methodologies can be adapted to different political environments, considering their unique actors, institutions, and issues.

#### Detection models

To detect political content, we used three groups of techniques: CSML, DL, and dictionary-based ones. The trained CSML models and dictionaries are available via the OSF repository (https://osf.io/e8xtb/?view_only=0c58144e1769492cb32dd2d650062534). The trained DL models are available via the Harvard Dataverse repository (https://dataverse.harvard.edu/dataset.xhtml?persistentId=doi:10.7910/DVN/8Q5FPE). We were interested in the low-cost implementation of these techniques, in particular in terms of minimising the resources required to obtain data for model training and conducting the actual training (e.g. time- and processing-wise). Such an interest is attributed to our assumption that many academic projects might have limited financial/time resources for in-house NLP technique development; hence, we wanted to compare the performance of detection techniques under these unfavourable circumstances.

For CSML, we used five models: logistic regression (LR), passive aggressive (PA), Bernoulli naive Bayes (BNB), multinomial naive Bayes (MNB), and stochastic gradient descent (SGD). These models differ in complexity, with some being based on simple Bayesian probabilistic modelling (e.g. BNB) and others relying on more advanced incremental learning principles (e.g. PA). All CSML models were trained using the Scikit-Learn package for Python [47].

For DL, we used three models: CNN, LSTM, and BERT. CNNs have low computational costs compared with other types of DL-based models and focus on high-level features, while LSTMs place major emphasis on term sequences. Finally, BERT [30] is a transformer model characterised by high computational costs but also more advanced capabilities for processing sequential data, along with an extensive awareness of contextual relationships between words attributed to it being pretrained on a large text corpus. These models, especially BERT, are commonly used for detecting content associated with the different types of societal phenomena (for examples, see [48, 49]). While there are certainly other DL models which can be considered, for feasibility reasons we had to focus on a small selection of models which we find particularly relevant for politics-related content detection.

To train the CNN and LSTM models, we used the Python Tensorflow library [50], whereas, for BERT, we relied on a fine-tuned model for the German language from HuggingFace [51]. Because of our interest in low-cost detection approaches, we used simple CNN and LSTM architectures (see Appendix 3 in S1 File) with five learning epochs and 256 embedding dimensions. For BERT, we used three epochs because of the higher computational costs of model training and tested a series of probabilities for the “political” label to be assigned. Based on the F1 scores achieved per probability (see Appendix 4 in S1 File), we opted for 0.15 probability, which resulted in the highest F1 score.

As training data for the CSML- and DL-based models, we used a set of 4,023 articles crawled from German and Swiss journalistic media (i.e. *Blick*, *Bild*, and *Süddeutsche Zeitung*). To minimise the efforts required to manually annotate the data, we relied on metadata-based annotation in the form of journalistic tags (i.e. news categories such as “politics” or “sport” [26]). These tags were used as labels to divide the crawled data into 1,523 political and 2,500 non-political articles, which were then used for model training based on an 80–20 train–test split. While the presence of such out-of-the-box annotation is a substantial advantage of using journalistic content as training data, it can also make the resulting detection models less effective for processing non-journalistic content.

For dictionaries, we used three models: Di-CAP, Di-LL, and Di-CAP-LL. The Di-CAP dictionary is made up of terms from the German codebook for the Comparative Agendas Project (CAP [52]). The codebook is used to label political themes in Germany (e.g. economy or foreign politics) and includes key terms related to them. We also added a theoretically conceptualised list of terms for topics underrepresented in the CAP codebook (i.e. elections and ecology), together with a list of political actors’ names in Germany/Switzerland (e.g. members of parliament) and G20/EU countries (e.g. presidents and vice-ministers).

The Di-LL dictionary is based on the same set of 4,023 journalistic articles that were used for the CSML- and DL-based models. The two subsets of articles—political and non-political—were transformed into bags of words. Then, we used log-likelihood keyword analysis [53] to identify terms that were overrepresented in the political subset. Following existing studies [54], we created two sub-dictionaries consisting of the top 100 and top 1,000 terms (according to log-likelihood scores) and then compared their performance across three validation datasets using the no-preprocessing option (see below). Based on the average F1 scores across the validation datasets, the 100-term option demonstrated better performance and was thus used in the study.

Finally, the Di-CAP-LL dictionary combines terms from the Di-CAP and Di-LL dictionaries with filtered-out duplicates. The assumption here is that the combined dictionary might outperform its components by bringing together the theoretically informed set of terms (Di-CAP) and the empirically driven set of terms that were most common in politics-related journalistic articles (Di-LL).

A major difference in using dictionaries compared with CSML- and DL-based techniques is that, by default, dictionaries do not provide a binary label (i.e. an indicator of whether the document is related to politics). Instead, dictionaries allow for the identification of the number of politics-related terms within a document that has then to be translated into a binary label. To address the possible variation in the length of documents coming from different platforms, we relied not on the absolute numbers but on the ratio between the number of politics-related unique terms to the overall number of unique terms per document.

After calculating all the ratios per validation dataset, we then used each of these ratios as a possible threshold for assigning a political label to all the documents in the respective validation dataset and calculated the resulting average F1 scores. Then, we chose the three thresholds that achieved the maximum F1 score for the three validation datasets (i.e. the one threshold per dataset) and applied each of these thresholds to all three datasets to identify a single threshold that would demonstrate the most consistent performance. This procedure was repeated for each dictionary-based technique and for each of the six modes of preprocessing. The complete list of optimal thresholds is provided in Appendix 5 in S1 File, but in most cases, the best performance was achieved, with the threshold of at least 0.5% of all unique terms in the document being present in the respective dictionaries.

#### Classification criteria

*Classification criteria for the training material*. Following the recommendation of previous work [14, 26], the criteria for the training material to be considered “political content” is determined by whether a given news article is categorised under the “politics” section on the media organisation’s website. We therefore use the knowledge and cues from media organisations to define political content.

*Classification criteria for the models*. The classification criteria for our models—whether they are dictionary-, CSML- or DL-based—are determined by the model’s prediction of the text being political. The exact criterion for the prediction depends on the model: for instance, for CSML- and DL-based models, the assignment of the label is binary and is based on the model’s training. By contrast, for the dictionary-based approaches, we calculated the threshold of a certain number of politics-related terms that correspond to the higher likelihood of a text being related to the politics (see the description of the procedure in the previous section).

*Classification criteria for the validation dataset*. The criteria used to classify our validation datasets as political or not were based on manual annotation. Human coders were provided with a codebook (Appendix A1 in S1 File) that reflects our broad definition of politics as including processes and political procedures (politics), form, structures, and institutional aspects (polity), and the content of political disputes (policy). Therefore, coders were asked whether each of the content items deals with either of these politics-related aspects to be considered political content.

### Data preprocessing

We lowercased all words in the training and validation datasets to avoid potential inconsistencies and removed punctuation using the Python String package [55]. Then, we compared the models’ performance in six preprocessing approaches: (1) no additional preprocessing; (2) stopword removal (using German stopwords from the Python Natural Language Toolkit (NLTK) library [56]); (3) stemming (using the Cistem stemmer from the NLTK library [56]); (4) lemmatisation (using German lemmatiser from the SpaCy library [57]); (5) stopword removal + stemming; and (6) stopword removal + lemmatisation. Altogether, this process resulted in 66 model combinations (11 models x 6 preprocessing approaches).

### Validation of detection models

To evaluate the models’ performance, we created three validation datasets: (1) a *test validation dataset* (TVD) made up of a subsample of training data (805 journalistic stories; 20% of the training sample); (2) a *diverse validation dataset* (DVD) made of 594 short (e.g. tweets) and long content pieces (e.g. articles from German right-wing outlets); and (3) a *web-tracking validation dataset* (WVD) consisting of 262 documents coming from the corpus of web-tracking data. The TVD was produced following the same principle as the training data (see above), whereas the DVD and WVD were manually annotated (see Appendix 1 in S1 File).

The three datasets were characterised by various degrees of content diversity as well as noise, defined as the “difference in the surface form of an electronic text from the intended, correct or original text” [12, p. 5]. The TVD and DVD had little noise because their content was crawled from a small selection of platforms and carefully parsed. In terms of content diversity, the TVD made only of news articles was the least diverse, whereas the DVD had more diversity. Finally, the WVD had the highest amount of noise, as well as the most content diversity, because its content came from a broad range of platforms to which a cross-platform HTML parser (based on the Selectolax Python library [58]) was applied. The necessity to rely on cross-platform HTML parsers is a major challenge associated with the use of web-tracking data (i.e. our use case), as well as the related problem of cross-platform automated content analysis. The use of cross-platform parsers results in a higher volume of noise associated with parsing errors (e.g. infection of organic texts with malparsed HTML tags), but the only alternative is the use of platform-specific parsers, which is not feasible when the data come from thousands of platforms.

To measure the models’ performance, we calculated the set of metrics commonly used in the NLP research—precision, recall, and F1 scores—for both predicted classes (i.e. political and non-political), together with the average values for these metrics. Precision is the ratio of true positive cases to the sum of true positives and false negatives, recall is the ratio of true positives to the sum of true positives and false positives, and the F1 score is the harmonic mean of precision and recall. For readability’s sake, we report in the next section only the political class and average F1 scores (for the full metrics, see Appendix 6 in S1 File).

It is important to note that no cross-validation was used when measuring the performance of the models, which made our observations about their performance less robust. This is a major limitation of the study that is attributed to the large number of models compared and the limited computational resources available, with the latter factor being particularly relevant for more computationally demanding techniques (e.g. the ones using BERT). Instead, we opted for a fixed train–test split to make the comparison between the models more consistent by ensuring that all models used the same data for training.

## Results

Table 1 demonstrates that the CSML-based models (passive-aggressive model) achieved the best performance on the TVD, with F1 scores for the political class reaching 0.89 (PA). The DL-based models (BERT) also showed high performance with F1 scores up to 0.86. Such a performance can be attributed to the TVD being the least noisy dataset, as well as the most similar to the data on which the CSML- and DL-based models were trained. The lower performance of the CNN model can be attributed to it being substantially simpler (and less computationally demanding) compared with LSTM and BERT. The dictionary-based models, in particular Di-CAP, showed acceptable results but performed worse than CSML- and DL-based models, which is an observation that aligns with earlier comparative NLP studies [27].

**Table 1:** Models’ performance on the test validation dataset (TVD). In this and the following tables, the highest performance values per preprocessing mode are bolded.

	No preprocessing	Stopword removal	Stemming	Stemming + stopword removal	Lemmatisation	Lemmatisation + stopword removal
Di-CAP	0.76 [0.79]	0.75 [0.77]	0.69 [0.71]	0.69 [0.70]	0.76 [0.78]	0.75 [0.77]
Di-LL	0.71 [0.71]	0.70 [0.70]	0.67 [0.67]	0.72 [0.74]	0.66 [0.69]	0.67 [0.69]
Di-CAP-LL	0.76 [0.77]	0.72 [0.73]	0.71 [0.71]	0.73 [0.75]	0.74 [0.77]	0.74 [0.75]
CSML [PA]	**0.88 [0.91]**	**0.89 [0.91]**	**0.88 [0.90]**	**0.88 [0.91]**	**0.89 [0.91]**	**0.89 [0.91]**
CSML [BNB]	0.86 [0.89]	0.86 [0.89]	0.85 [0.88]	0.86 [0.88]	0.88 [0.90]	0.88 [0.90]
CSML [MNB]	0.88 [0.90]	**0.89 [0.91]**	0.87 [0.89]	0.88 [0.90]	0.89 [0.91]	0.88 [0.90]
CSML [LR]	0.87 [0.89]	0.86 [0.88]	0.86 [0.89]	0.87 [0.89]	0.85 [0.88]	0.87 [0.89]
CSML [SGD]	0.83 [0.86]	0.86 [0.89]	0.86 [0.89]	0.85 [0.88]	0.86 [0.89]	0.85 [0.88]
DL [CNN]	0.73 [0.80]	0.82 [0.86]	0.49 [0.58]	0.83 [0.87]	0.82 [0.86]	0.83 [0.86]
DL [LSTM]	0.79 [0.83]	0.87 [0.90]	0.85 [0.88]	0.79 [0.83]	0.86 [0.88]	0.85 [0.88]
DL [BERT]	0.86 [0.88]	0.81 [0.82]	0.81 [0.83]	0.79 [0.79]	0.85 [0.87]	0.83 [0.84]

In terms of preprocessing, we observed close to no impact on the best-performing CSML-based models (i.e. a change in the range of 0.01–0.02 for the F1 scores) and little impact on the best-performing DL-based models (i.e. changes in the range of 0.02–0.06). While lemmatisation provided one of the best results, similar scores were also achieved with only stopword removal or no processing (e.g. for BERT). In the case of dictionaries, we observed a similar pattern with lemmatisation and no processing, which provided optimal results.

For the DVD (Table 2), the best performance was achieved by BERT (up to a 0.90 F1 score for the political class). The other DL- and CSML-based models showed a major drop in performance, which can be attributed to the increase in content diversity. We expect that additional fine-tuning (e.g. expansion of the network architecture or an increase in the number of learning epochs) would improve these models’ performance; however, under the condition of the non-fine-tuned low-cost implementation, BERT provided substantially better results. The second-best performance (0.77 F1 score for the political class) was achieved by Di-CAP-LL, which can be attributed to dictionary-based models being more robust when dealing with diverse content.

**Table 2:** Models’ performance on the diverse validation dataset (DVD).

	No preprocessing	Stopword removal	Stemming	Stemming + stopword removal	Lemmatisation	Lemmatisation + stopword removal
Di-CAP	0.75 [0.70]	0.75 [0.70]	0.75 [0.65]	0.69 [0.63]	0.75 [0.7]	0.76 [0.70]
Di-LL	0.66 [0.58]	0.66 [0.57]	0.64 [0.55]	0.63 [0.58]	0.56 [0.52]	0.56 [0.51]
Di-CAP-LL	0.75 [0.68]	0.76 [0.66]	0.77 [0.67]	0.73 [0.66]	0.73 [0.66]	0.56 [0.51]
CSML [PA]	0.49 [0.51]	0.47 [0.50]	0.52 [0.53]	0.50 [0.51]	0.50 [0.51]	0.49 [0.51]
CSML [BNB]	0.36 [0.43]	0.36 [0.43]	0.38 [0.44]	0.44 [0.48]	0.38 [0.44]	0.43 [0.47]
CSML [MNB]	0.69 [0.65]	0.69 [0.65]	0.63 [0.60]	0.68 [0.64]	0.67 [0.63]	0.71 [0.67]
CSML [LR]	0.44 [0.48]	0.41 [0.46]	0.46 [0.49]	0.43 [0.48]	0.44 [0.48]	0.42 [0.47]
CSML [SGD]	0.65 [0.61]	0.37 [0.43]	0.59 [0.57]	0.62 [0.58]	0.58 [0.56]	0.66 [0.61]
DL [CNN]	0.15 [0.30]	0.27 [0.37]	0.29 [0.34]	0.36 [0.43]	0.32 [0.41]	0.35 [0.42]
DL [LSTM]	0.53 [0.53]	0.44 [0.48]	0.54 [0.55]	0.54 [0.54]	0.52 [0.53]	0.53 [0.53]
DL [BERT]	**0.85 [0.78]**	**0.90 [0.83]**	**0.87 [0.79]**	**0.89 [0.81]**	**0.86 [0.79]**	**0.90 [0.84]**

Similar to the TVB, the impact of preprocessing was limited for the DVD. For BERT, we observed changes in the range of 0.01–0.05, with the best results achieved by stopword removal and lemmatisation with stopword removal. A similar effect was observed for the dictionary-based models, where stopword removal or lemmatisation/stemming enabled the best performance. The largest preprocessing-based fluctuations were observed for CNN, where the addition of more complex forms of preprocessing led to a major increase (i.e. up to 0.21) in the F1 score for the political class; however, the overall performance of the model remained too low to be usable.

Finally, for the noisiest and most diverse validation dataset (WVD; Table 3), we observed the best performance from the dictionary-based approach. The F1 scores for these approaches reached 0.81 (Di-CAP) when used with lemmatisation or lemmatisation with stopword removal for the political class, as contrasted to the 0.63 F1 score for the best DL-based model (BERT). Similar to the TVD, the addition of the log-likelihood sub-dictionary usually worsened the performance of the CAP-based sub-dictionary (except for the case when the Di-CAP-LL was combined with stemming); the latter observation can be explained by the Di-LL bringing additional semantic noise that is more detrimental for highly diverse data.

**Table 3:** Models’ performance on the web-tracking validation dataset (WVD).

	No preprocessing	Stopword removal	Stemming	Stemming + stopword removal	Lemmatisation	Lemmatisation + stopword removal
Di-CAP	**0.79 [0.83]**	**0.79 [0.83]**	0.62 [0.72]	**0.72 [0.77]**	**0.81 [0.85]**	**0.81 [0.85]**
Di-LL	0.68 [0.75]	0.65 [0.73]	0.59 [0.66]	0.61 [0.66]	0.63 [0.70]	0.63 [0.70]
Di-CAP-LL	0.73 [0.79]	0.76 [0.81]	**0.63 [0.72]**	0.67 [0.75]	0.78 [0.83]	0.78 [0.82]
CSML [PA]	0.47 [0.65]	0.44 [0.63]	0.48 [0.65]	0.42 [0.62]	0.45 [0.63]	0.42 [0.61]
CSML [BNB]	0.22 [0.51]	0.30 [0.55]	0.28 [0.54]	0.23 [0.51]	0.22 [0.51]	0.22 [0.51]
CSML [MNB]	0.44 [0.63]	0.44 [0.63]	0.34 [0.57]	0.46 [0.64]	0.39 [0.60]	0.43 [0.62]
CSML [LR]	0.50 [0.67]	0.49 [0.66]	0.50 [0.67]	0.49 [0.66]	0.52 [0.68]	0.48 [0.66]
CSML [SGD]	0.48 [0.63]	0.47 [0.65]	0.41 [0.59]	0.38 [0.57]	0.51 [0.66]	0.52 [0.66]
DL [CNN]	0.12 [0.45]	0.28 [0.54]	0.30 [0.51]	0.38 [0.59]	0.34 [0.57]	0.46 [0.64]
DL [LSTM]	0.1 [0.54]	0.36 [0.58]	0.37 [0.59]	0.46 [0.61]	0.37 [0.58]	0.24 [0.51]
DL [BERT]	0.32 [0.56]	0.58 [0.71]	0.54 [0.67]	0.63 [0.71]	0.45 [0.63]	0.63 [0.72]

From the preprocessing point of view, lemmatisation again delivered the best results, followed by stopword removal and the absence of preprocessing. The small variation between the best-performing scores (i.e. 0.02) suggests that the use of more computationally demanding options might not be justified, especially when dealing with large volumes of cross-platform data. Interestingly, stemming led to a substantial performance drop (0.62 compared with 0.79 with no preprocessing for Di-CAP), which may be due to its creation of artificial noise (e.g. by creating unwanted ambiguities caused by the resolution of words to their stems).

## Discussion

The increase in the volume and diversity of political content available online prompts the need for cross-platform approaches for its detection. In this paper, we discuss various ways to address this problem by comparing three groups of detection techniques—dictionary, CSML, and DL-based ones—and examining the impact of preprocessing on their performance.

Our results show that while the CSML- and DL-based models demonstrated solid performance on content similar to the type they were trained on (i.e. journalistic articles in our case), their effectiveness dropped for more diverse content. The major exception here is BERT, which highlights the ability of more computationally demanding transformer models to achieve high performance on data coming from a broad range of platforms; this finding aligns with the existing evaluations of transformers being the state-of-the-art approach in the field of NLP [59]. However, for web-tracking data with a high amount of noise, even BERT’s performance turned out to be low.

While dictionaries did not show the best performance on less noisy data, for web-tracking data, they outperformed more complex techniques. There may be two reasons for this. First, the underlying principle of the dictionary (i.e. word matching) might be more fitting for detection tasks with a specific focus (e.g. phenomena associated with a concrete set of actors) and less vulnerable to data noise (e.g. HTML artefacts left after parsing). Second, the high scores of Di-CAP (i.e. dictionary combining actor/institution names and CAP terms) can be attributed to it being manually verified (and, hence, more fitting for the task) than journalistic tag-based datasets used for training other techniques. The latter interpretation is supported by the drop in the performance of the combined dictionary (i.e. Di-LL-CAP), which may be due to the LL dictionary bringing additional noise. This difference highlights the fact that the combination of dictionaries does not automatically lead to performance improvement.

The high performance of Di-CAP does not mean that dictionary-based techniques always outperform CSML- and DL-based techniques. However, under the condition of limited development resources, the reuse of an existing dictionary with the possibility of additional augmentations might provide better results than reliance on metadata-based labels (e.g. journalistic tags) for CSML- and DL-based models. With enough resources available, we expect a diverse corpus of manually annotated training data to potentially outperform dictionaries, as has been the case with other automated content analysis tasks [27]. At the same time, dictionary-based approaches are also often distinguished by higher explainability and transparency regarding decision-relevant elements of the detection process (as contrasted by different machine learning-based approaches) that can make it easier to understand which factors have contributed to the accuracy of individual approach in training or in the context of a particular prediction.

Lastly, when comparing different approaches discussed in the study, it is important to consider how they differ in terms of computational complexity. For instance, dictionary-based approaches involve simple string matching, so the computational complexity is low, whereas machine learning-based (especially DL-based) approaches are more computationally complex. To illustrate this difference, we can consider the amount of time required to deal with the same task: while dictionaries and supervised machine learning models took 0.125 and 0.328 seconds per detection task, respectively, this number increases significantly for the LSTM model (35 seconds per detection task), and even further for the BERT model (139.59 seconds per detection task).

These results have several implications for the use of automated content analysis in political communication research. First, they show that designing cross-platform detection approaches is possible, even though the process of doing so remains rather challenging. The success of such an endeavour depends on the robustness of the chosen approach and the amount of available resources (either in the form of in-house development capacities or third-party assets made accessible by the research community). The combination of these two factors makes dictionary-based approaches particularly appealing: not only might they be less subjected to data noise (especially in the case of cross-platform forms of passive data collection [13]), but the procedure for asset reuse is also more intuitive for dictionaries compared with CSML- or DL-based models. It is, however, important to remember that the correct identification of political content across platforms is often the first step in a deeper analysis of discourse around politics and policy. It is likely that substantial policy discussions about migration (for example) will use significantly different language than toxic debates, making use of hate speech around the same issue. Therefore, future work should enhance this first step of correctly identifying political material across platforms with other NLP techniques that allow for a more nuanced separation of political language and its implications.

Second, our results resonate with earlier calls [34] for the extensive validation of automated content analysis techniques. While some CSML and DL models showed high performance on the TVD, it substantially worsened on less familiar and more diverse content and dropped even further on data containing a high volume of noise. These observations stress the importance of utilising more than one validation dataset for measuring the performance of the NLP techniques used to study communication phenomena, together with making these validation datasets diverse (i.e. by including content coming from different platforms), especially when aiming to make these techniques work across platforms.

Finally, our study offers insights into the impact of preprocessing on different NLP techniques. Specifically, it suggests that there is little difference between the models’ performance under the conditions of no preprocessing and when more complex preprocessing modes are used. This observation is particularly relevant for more computationally demanding modes (e.g. lemmatisation), where marginal increases in performance do not necessarily justify the time and resource costs needed to apply them to large datasets. This leads us to a conclusion similar to those of earlier NLP studies [35], in that stopword removal is often an optimal mode of preprocessing because it allows for a performance increase at a relatively small cost.

Our study acknowledges the growing importance of analysing social media content, and other online sources, for understanding public discourse, particularly in political communication. Platforms like Twitter offer rich, unstructured data that can provide valuable insights into public opinions and behaviour, especially in the context of policy responses and citizen interactions with officials [60]. However, these platforms often exhibit biases in user demographics and opinions, which may limit the representativeness of the data and its generalisability [61, 62]. This limitation underscores the need for our detection techniques to not only be robust across various content types but also to consider the inherent features of content coming from specific platforms. Our findings suggest that while dictionary-based approaches offer certain advantages in handling noisy data, there is also a critical need for diverse and representative training data to enhance the performance and applicability of more complex models like CSML and DL-based techniques. Integrating insights from diverse datasets and accounting for platform-related content features are essential steps towards developing more accurate and generalisable automated content analysis methods. This alignment with broader research perspectives highlights the importance of situating our contributions within the ongoing discourse about the representativeness and reliability of platform data in political communication research.

### Limitations

This paper is not without limitations. First, we focused on low-cost implementations of the detection techniques without using additional fine-tuning or training resources (e.g. increases in the number of epochs for DL-based models). Such a focus is attributed to the expectation that many academic-based projects would have limited resources for implementing such techniques, but it should be taken into consideration that a comparison of high-cost implementations might change the results. Second, the training data for the CSML- and DL-based techniques (as well as the Di-LL dictionary) were made exclusively of journalistic articles. While this choice allows for the utilisation of metadata-based labels and, thus, avoids the need for manual labelling of the training data, it results in the bias of models towards specific formats of content (i.e. journalistic articles) and makes it harder for models other than BERT to deal with more diverse and noisy data. Future research could benefit from relying on more diverse sets of training data, especially when dealing with cross-platform content detection tasks. Third, because of the large number of models compared, together with the limited computational resources, we did not use cross-validation to make the evaluations of our models’ performance more robust. For future research, it is important to integrate cross-validation into the process of performance evaluation to obtain more generalisable insights.

## Conclusions

In this article, we examined the performance of different methodological approaches and modes of preprocessing regarding the task of low-cost automated detection of politics-related content in textual data coming from different platforms. Our examination shows that preprocessing has limited impact on the performance, at least for the best-performing models. In terms of approaches, we found that the best results for less noisy data are achieved by DL- and CSML-based models, whereas for more noisy data dictionary-based approaches can show a more robust performance.

## Supporting information

S1 File Appendix for the "Panning for gold: Comparative analysis of cross-platform approaches for automated detection of political content in textual data" article. The appendix contains information about the definition of politics-related content and procedure for manually labelling it for validation datasets, the use case of the study, supplementary information for dictionary- and DL-based approaches, and the complete set of performance metrics for the models.
(DOCX)

## References

[1] van Aelst P, et al. Political Communication in a High-Choice Media Environment: A Challenge For Democracy? *Annals of the International Communication Association*. 2017;41: 3–27.

[2] Mukerjee S, Majó-Vázquez S, González-Bailón S. Networks of Audience Overlap in the Consumption of Digital News. *Journal of Communication*. 2018;68: 26–50.

[3] Prior M. The Challenge of Measuring Media Exposure: Reply to Dilliplane, Goldman, and Mutz. *Political Communication*. 2013;30: 620–634.

[4] Christner C, et al. Automated Tracking Approaches for Studying Online Media Use: A Critical Review and Recommendations. *Communication Methods and Measures*. 2021; doi: 10.1080/19312458.2021.1907841

[5] Makhortykh M, et al. We Are What We Click: Understanding Time and Content-Based Habits of Online News Readers. *New Media & Society*. 2021;23: 2773–2800.

[6] Wojcieszak M, et al. No Polarization from Partisan News: Over-Time Evidence from Trace Data. *The International Journal of Press/Politics*. 2021; doi: 10.1177/19401612211047194

[7] Dvir-Gvirsman S, Tsfati Y, Menchen-Trevino E. The Extent and Nature of Ideological Selective Exposure Online: Combining Survey Responses with Actual Web Log Data from the 2013 Israeli Elections. *New Media & Society*. 2016;18: 857–877.

[8] Stier S, et al. Populist Attitudes and Selective Exposure to Online News: A Cross-Country Analysis Combining Web Tracking and Surveys. *The International Journal of Press/Politics*. 2020;25: 426–446.

[9] de León E, Trilling D. A Sadness Bias in Political News Sharing? The Role of Discrete Emotions in the Engagement and Dissemination of Political News on Facebook. *Social Media + Society*. 2021;7: 1–12.

[10] de Mello Araújo E, Ebbelaar D. Detecting Dutch Political Tweets: A Classifier Based on Voting System Using Supervised Learning. In: *Proceedings of the 10th International Conference on Agents and Artificial Intelligence*, 2018 Jan 16–18; Funchal, Portugal. Setúbal: SCITEPRESS; 2018 pp. 462–469.

[11] Ho Y, Pepyne D. Simple Explanation of the No-Free-Lunch Theorem and its Implications. *Journal of Optimization Theory and Application*s. 2002;115: 549–570.

[12] Agarwal S, et al. How Much Noise is Too Much: A Study in Automatic Text Classification. In: *Seventh IEEE International Conference on Data Mining*, 2007 Oct 28–31; Omaha, USA. Piscataway: IEEE; 2007 pp. 3–12.

[13] Stier S, et al. Integrating Survey Data and Digital Trace Data: Key Issues in Developing an Emerging Field. *Social Science Computer Review*. 2020;38: 503–516.

[14] Guess A. The Consequences of Online Partisan Media. *Proceedings of the National Academy of Sciences*. 2021;118: 1–8. doi: 10.1073/pnas.2013464118 33782116 PMC8040813

[15] de León E, et al. News, Threats, and Trust: How COVID-19 News Shaped Political Trust, and How Threat Perceptions Conditioned This Relationship. *The International Journal of Press/Politics*. 2022; doi: 10.1177/19401612221087179

[16] Merten L, et al. News Won’t Find Me? Exploring Inequalities in Social Media News Use with Tracking Data. *International Journal of Communication*. 2022;16: 1127–1147.

[17] Benoit K, et al. Crowd-Sourced Text Analysis: Reproducible and Agile Production of Political Data. *American Political Science Review*. 2016;110: 278–295.

[18] Ophir Y, et al. News Media Framing of Social Protests around Racial Tensions during the Donald Trump Presidency. *Journalism*. 2021; doi: 10.1177/14648849211036622

[19] Barberá P, et al. Automated Text Classification of News Articles: A Practical Guide. *Political Analysis*. 2021;29: 19–42.

[20] Dun L, Soroka S, Wlezien C. Dictionaries, Supervised Learning, and Media Coverage of Public Policy. *Political Communication*. 2021;38: 140–158.

[21] Sang E, Bos J, Predicting the 2011 Dutch Senate Election Results with Twitter. In: *Proceedings of the Workshop on Semantic Analysis in Social Media*; 2012. Stroudsburg: ACL; 2012, pp. 53–60.

[22] Heiss R, Matthes J. Stuck in a Nativist Spiral: Content, Selection, and Effects of Right-Wing Populists’ Communication on Facebook. *Political Communication*. 2020;37: 303–328.

[23] Boumans J, Trilling D. Taking Stock of the Toolkit: An Overview of Relevant Automated Content Analysis Approaches and Techniques for Digital Journalism Scholar. *Digital Journalism*. 2016;4: 8–23.

[24] Hamoud A, et al. Classifying Political Tweets Using Naïve Bayes and Support Vector Machines. In: *International Conference on Industrial*, *Engineering and Other Applications of Applied Intelligent Systems*, 2018 Jun 25–28; Montreal, Canada. Cham: Springer; 2018 pp. 736–744.

[25] de León E, Vermeer S, Trilling D. Electoral News Sharing: A Study of Changes in News Coverage and Facebook Sharing Behaviour During the 2018 Mexican Elections. *Information*, *Communication & Society*. 2021; doi: 10.1080/1369118X.2021.1994629

[26] Stier S, et al. Post Post-Broadcast Democracy? News Exposure in the Age of Online Intermediaries. *American Political Science Review*. 2021;116: 768–774.

[27] Van Atteveldt W, van der Velden M, Boukes M. The Validity of Sentiment Analysis: Comparing Manual Annotation, Crowd-Coding, Dictionary Approaches, and Machine Learning Algorithms, *Communication Methods and Measures*. 2021;15: 121–140.

[28] Chang C, Masterson M. Using Word Order in Political Text Classification with Long Short-Term Memory Models. *Political Analysis*. 2020;28: 395–411.

[29] Luan Y, Lin S. Research on Text Classification Based on CNN and LSTM. In: *2019 IEEE International Conference on Artificial Intelligence and Computer Applications*; 2019 Mar 29–31; Dalian, China; Piscataway: IEEE; 2019 pp. 352–355.

[30] Devlin J, et al. Bert: Pre-Training of Deep Bidirectional Transformers for Language Understanding. In: *Proceedings of the 2019 Conference of the North American Chapter of the Association for Computational Linguistics*: *Human Language Technologies*, 2019 Jun 2–7; Minneapolis, USA. Stroudsburg: ACL; 2019 pp. 4171–4186.

[31] Kim B, Park J, Suh J. Transparency and Accountability in AI Decision Support: Explaining and Visualizing Convolutional Neural Networks for Text Information. *Decision Support Systems*. 2020;134: 1–11.

[32] Rao A, Spasojevic N. Actionable and Political Text Classification Using Word Embeddings and LSTM. Preprint. arXiv:1607.02501 [Preprint]. 2016 [cited 2023 Feb 11]. Available from: https://arxiv.org/abs/1607.02501

[33] Kulkarni V et al. Multi-View Models for Political Ideology Detection of News Articles. In: *Proceedings of the 2018 Conference on Empirical Methods in Natural Language Processing*; 2018 Oct 31-Nov 4; Brussels, Belgium. Stroudsburg: ACL; 2018 pp. 3518–3527.

[34] Grimmer J, Stewart B. Text as Data: The Promise and Pitfalls of Automatic Content Analysis Methods for Political Texts. *Political Analysis*. 2013;21: 267–297.

[35] HaCohen-Kerner Y, Miller D, Yigal Y. The Influence of Preprocessing on Text Classification Using a Bag-of-Words Representation. *PloS one*. 2020;15: 1–22. doi: 10.1371/journal.pone.0232525 32357164 PMC7194364

[36] Denny M, Spirling A. Text Preprocessing for Unsupervised Learning: Why It Matters, When It Misleads, and What To Do About It. *Political Analysis*. 2018;26: 168–189.

[37] Bounabi M, Moutaouakil K, Satori K. A Comparison of Text Classification Methods Using Different Stemming Techniques. *International Journal of Computer Applications in Technology*. 2019;60: 298–306.

[38] Toman M, Tesar R, Jezek K. Influence of Word Normalization on Text Classification, *Proceedings of InSciT*. 2006;4: 354–358.

[39] HaCohen-Kerner Y, et al. Topic-Based Classification Through Unigram Unmasking, *Procedia Computer Science*. 2018;126: 69–76.

[40] Méndez J, et al. Tokenising, Stemming and Stopword Removal on Anti-Spam Filtering Domain. In: *Conference of the Spanish Association for Artificial Intelligence*; 2005 Nov 16–18; Santiago de Compostela, Spain. Berlin: Springer; 2005 pp. 449–458.

[41] Gonçalves C, et al. The Impact of Pre-Processing on the Classification of MEDLINE Documents. In: *Proceedings of the 10th International Workshop on Pattern Recognition in Information Systems*, Funchal, Portugal. Setúbal: SCITEPRESS; 2010 pp. 53–61.

[42] El Kah A, Zeroual I. The Effects of Preprocessing Techniques on Arabic Text Classification. *International Journal of Advanced Trends in Computer Science and Engineering*. 2021;10: 1–12.

[43] Song F, Liu S, Yang J. A Comparative Study on Text Representation Schemes in Text Categorization. *Pattern Analysis and Applications*. 2005;8: 199–209.

[44] Maulana I, Maharani W. Disaster Tweet Classification Based on Geospatial Data Using the BERT-MLP Method. In: *9th International Conference on Information and Communication Technology*; 2021 Aug 3–5; Yogyakarta, Indonesia. Piscataway: IEEE; 2021 pp. 76–81.

[45] Konstantinov A, Moshkin V, Yarushkina N. Approach to the Use of Language Models BERT and Word2vec in Sentiment Analysis of Social Network Texts. In: Dolinina O et al., editors. *Recent Research in Control Engineering and Decision Making*. Cham: Springer; 2020, pp. 462–473.

[46] Nohlen D, Bernhard T. Politisches System. In: Nohlen D, Grotz F, editors. Kleines Lexikon der Politik. Bundeszentrale für Politische Bildung; 2011. pp. 487–489.

[47] Pedregosa F, et al. Scikit-learn: Machine learning in Python, *The Journal of Machine Learning Research*. 2011;12: 2825–2830.

[48] Bilbao-Jayo A, Almeida A. Political discourse classification in social networks using context sensitive convolutional neural networks. In Proceedings of the Sixth International Workshop on Natural Language Processing for Social Media; 2018. Stroudsburg: ACL; 2018, pp. 76–85.

[49] Busioc C et al. What are the latest fake news in romanian politics? an automated analysis based on bert language models. In Proceedings of the 6th International Conference on Smart Learning Ecosystems and Regional Development; 2022. Singapore: Springer; 2022, pp. 201–212.

[50] Abadi M, et al. Tensorflow: Large-Scale Machine Learning on Heterogeneous Distributed Systems. arXiv:1603.04467 [Preprint]. 2016 [cited 2023 Feb 11]. Available from: https://arxiv.org/abs/1603.04467

[51] HuggingFace. *Bert-base-german-cased* [cited 2023 January 29]. Available from: https://huggingface.co/bert-base-german-cased, 2020

[52] Bevan S. Gone Fishing: The Creation of the Comparative Agendas Project Master Codebook. In: Baumgartner F, Breunig C, Grossman E, editors. *Comparative Policy Agendas*: *Theory*, *Tools*, *Data*. Oxford: Oxford University Press; 2019. pp. 17–34.

[53] Pojanapunya P, Todd RW. Log-Likelihood and Odds Ratio: Keyness Statistics for Different Purposes of Keyword Analysis. *Corpus Linguistics and Linguistic Theory*. 2018;14: 133–167.

[54] de Schryver G. Trends in Twenty-Five Years of Academic Lexicography. *International Journal of Lexicography*. 2012;25: 464–506.

[55] String—Common String Operations. 2023 [cited 2023 January 29]. Available from: https://docs.python.org/3/library/string.html

[56] Bird S, Klein E, Loper E. *Natural Language Processing with Python*: *Analyzing Text with the Natural Language Toolkit*. Sebastopol: O’Reilly Media; 2009.

[57] Honnibal M et al. 2020. spaCy: Industrial-strength Natural Language Processing in Python [Preprint]. 2020. [cited 2023 Feb 11]. Available from: doi: 10.5281/zenodo.1212303

[58] Golubin A. *Selectolax*. 2023 [cited 2023 January 29]. Available from: https://github.com/rushter/selectolax

[59] Biggiogera J, et al. BERT Meets LIWC: Exploring State-of-the-Art Language Models for Predicting Communication Behavior in Couples’ Conflict Interactions. In: *Companion Publication of the 2021 International Conference on Multimodal Interaction*, 2021 Oct 18–22; Montreal, Canada. New York: ACM; 2021 pp. 385–389.

[60] Sainz-Santamaria J et al. Contesting views on mobility restrictions in urban green spaces amid COVID-19—Insights from Twitter in Latin America and Spain. Cities. 2023;132: 1–17. doi: 10.1016/j.cities.2022.104094 36407936 PMC9648905

[61] Barbera P, Rivero G. Understanding the political representativeness of Twitter users. Social Science Computer Review. 2015;33: 712–729.

[62] Gómez JC et al. Predicción automática del nivel educativo en usuarios de Twitter en méxico. Realidad, datos y espacio. Revista internacional de estadística y geografía. 2021;12: 48–61.

