# Supplementary material for: Panning for gold: Comparative analysis of cross-platform approaches for automated detection of political content in textual data
Source: PLoS One. 2024 Nov 18;19(11):e0312865. doi: 10.1371/journal.pone.0312865 (PMC11573140; doi:10.1371/journal.pone.0312865)
Supplement: S1 File — The appendix contains information about the definition of politics-related content and procedure for manually labelling it for validation datasets, the use case of the study, supplementary information for dictionary- and DL-based approaches, and the complete set of performance metrics for the models. (DOCX) [file pone.0312865.s001.docx]

**Appendix**

**Appendix A1: Definition of politics-related content and procedure for manually labelling**  **it for validation datasets**

Our understanding of politics-related information is based on three dimensions that include (1) processes and political procedures (politics); (2) form, structures, and institutional aspects (polity); and (3) the content of political disputes (policy) [60]. When developing the codebook to label the content used to create the validation datasets for our detection approaches, we assumed the piece of content contained politics-related information if the respective piece of content related to at least one of the above-mentioned dimensions.

To prepare the two validation datasets for the classification of political content (i.e. DVD and WVD; the labelling of the last validation dataset [TVD] was based on the metadata in the form of journalistic tags), the coders manually labelled two sets of documents (for a summary of this process, see Table A1.1 below). The first set consisted of 594 documents made of short (e.g. Twitter and Telegram posts by politicians and journalists) and longer documents (articles from German legacy media, such as *Süddeutsche Zeitung*, and right-wing outlets, like *Journalistenwatch*) crawled online. The second set consisted of 262 documents randomly sampled from the web-tracking data.

Table A1.1 Summary of the procedures for preparing the validation datasets

| Dataset name | Size | Source | Preparation | Coding validation |
| --- | --- | --- | --- | --- |
| Test validation dataset (TVD) | 805 documents | Journalistic articles from *Blick*, *Bild*, and *Süddeutsche Zeitung* | No manual labelling; journalistic metadata used as labels | No validation |
| Diverse validation dataset (DVD) | 594 documents | Short (Twitter and Telegram posts by politicians and journalists) and longer documents (articles from German legacy media, such as *Süddeutsche* Zeitung, and right-wing outlets, like *Journalistenwatch*) crawled online | Four coders | 10% of the dataset used for reliability check: Cohen’s Kappa = .86 (political actor) and Cohen's Kappa = .68 (political topic); the complete dataset was checked and consensus-coded by two experts |
| Web-tracking validation dataset (WVD) | 262 documents | A random sample of web-tracking data | One coder | For all cases where the single coder was not sure, the two PIs reviewed, discussed and consensus-coded the material |

Each document in the two datasets (DVD and WVD) was coded according to the following three variables: (1) POLITICAL ACTOR, POLITICAL INSTITUTIONS, AND PRINCIPLES (i.e. *Are political actors mentioned in the text? Does the text mention political institutions?*); (2) POLITICAL TOPICS (i.e. *Are political topics/issues mentioned in the text?*); and (3) OTHER POLITICAL SUBJECT AREA. A detailed description of the variables is provided below; the document was classified as political if at least one of these variables was present in the document.

**Variable: POLITICAL ACTOR, POLITICAL INSTITUTIONS, AND PRINCIPLES**

***Are political actors mentioned in the text? Does the text mention political institutions?***

0 = no

1 = yes

*Municipal/regional/national international political actors such as:*

- (Members of) governments (e.g. government, president, chancellor, or minister)
- Opposition (e.g. opposition leader or opposition parliamentary group)
- Parliamentarian (e.g. member of the Bundestag or Nationalrat)
- Politician
- Party (e.g. party leader or members)
- Administration (e.g. ministry)

*Politically active citizens who take clear political action, such as:*

- Lobbying specific issues
- Formulating demands placed on politicians
- Organis ing demonstrations

*Entire state as political actors (i.e. local/regional/national/international political institutions and principles) such as:*

- Constitution
- Political institutions (e.g. parliament, Council of Europe, European Union, or courts of law)
- Laws and agreements
- Political culture (e.g. political attitudes of citizens)

**Variable: POLITICAL TOPICS**

***Are political topics/issues mentioned in the text?***

0 = no

1 = yes

*Political topics/issues at the municipal/regional/national/international level:*

- Working conditions and labour market
- Foreign and domestic trade
- Banks and finances
- Education
- Civil rights and liberties and minority rights
- German reunification
- Energy
- Family and social affairs
- Health care
- International relations and foreign aid
- Agriculture
- Mobility, transport, and traffic
- Public administration
- Political campaigns, referendums, initiatives, and votes
- Law and crime
- Environment
- Defence
- Water management
- Housing, construction, and spatial planning
- Economy
- Science and technology
- Other subject areas, if applicable: the subject is to be named in the variable ‘OTHER POLITICAL SUBJECT AREAS’

A. Content related to these subject areas was considered political if it presented problems/proposed solutions from a perspective relevant to a population group or society as a whole (not just to an individual).

B. Political issues could be described at different stages of the process:

- As a pure description of the problem (without a policy demand or action)
- As a demand for policy (still without action)
- As political discussions or actions

**Variable: OTHER POLITICAL SUBJECT AREAS**

*Information deals with political content in a subject area that was not mentioned in the variable ‘POLITICAL TOPICS’:*

- Naming of the subject area

**Appendix A2: Use case**

Our interest in developing cross-platform approaches for detecting politics-related information is related to the specific requirements of our use case that deals with web-tracking data. Similar to other web-tracking studies relying on a large number of participants (e.g. Stier et al. 2020), we acquired data from a sample of participants (N = 1,149) from Germany and Switzerland who were recruited via a market research company and subsequently installed web-tracking software on their desktop browsers. During the two web-tracking rounds in the spring and autumn of 2020, the software extracted the HTML content appearing in the browsers and then transferred it to the remote server by following the screen-scraping principle (for more information, see Christner et al. 2021).

To protect the participants’ privacy, we used a denylist (i.e. a list of websites visits to which were not captured, which in this case included healthcare-related websites, online banking portals, commercial websites, and pornography), as well as customised filters for social media platforms (i.e. to avoid capturing personally identifiable data as well as private data). The remaining visits were all captured, resulting in over four million HTML pages recorded from almost 90,000 unique domains. Such diversity makes it infeasible to develop platform-specific detection solutions capable of accounting for the distinct format and style of textual data associated with a specific platform. Hence, to be able to detect politics-related content in these multi-platform data, as well as to realise possibilities provided by these data (e.g. by looking at how the long tail of information consumption affects individual engagements with politics-related information), we needed a cross-platform means of automated detection.

**Appendix A3: DL-based model architectures**

To train the CNN- and LSTM-based models, we used tokenis ers with the 120,000 most common words in the training data. We used a batch size of 256 with five training epochs for each model, with a binary cross entropy function for measuring the loss during the model training and a Nadam optimiser. For both the CNN and LSTM models, we used sequential architectures with the composition of layers described in Tables A3.1 and A3.2.

Table A3.1 Composition of layers for the CNN network

| Layer | Activation |
| --- | --- |
| Embedding | None |
| 1D convolution layer | ReLU |
| Global max pooling for 1D data | None |
| Dropout layer | None |
| Dense layer | ReLU |
| Dropout layer | None |
| Dense layer | Sigmoid |

Table A3.2 Composition of layers for the LSTM network

| Layer | Activation |
| --- | --- |
| Embedding | None |
| Bidirectional LSTM | None |
| Global max pooling for 1D data | None |
| Dropout layer | None |
| Dense layer | ReLU |
| Dropout layer | None |
| Dense layer | Sigmoid |

**Appendix A4: Probability of assigning the “political” label by BERT models**

BERT allows for the specification of the threshold of the minimal probability of a specific label (e.g. “political”) being assigned to the document to which the model is applied. The lower this probability is, the less conservative the prediction, and vice versa. To identify the optimal probability, we tested different thresholds (i.e. 5%, 10%, 15%, 20%, 25%, 30%, 35%, and 40%) for the three validation datasets using the average F1 score as a performance measurement. The results of the testing process are provided in Table A4.1; based on the comparison of the aggregated F1 scores across all three datasets, we opted for a 15% probability that showed the highest performance.

**Table A4.1** Performance of BERT models per probability threshold (average F1 scores)

|  | Probability / preprocessing | 0.05 | 0.1 | 0.15 | 0.2 | 0.25 | 0.3 | 0.35 | 0.4 |
| --- | --- | --- | --- | --- | --- | --- | --- | --- | --- |
| TVD | No-pre | 0.82 | 0.86 | 0.88 | 0.88 | 0.88 | 0.89 | 0.88 | 0.88 |
|  | Stop | 0.74 | 0.79 | 0.82 | 0.84 | 0.85 | 0.87 | 0.88 | 0.89 |
|  | Ste | 0.73 | 0.80 | 0.83 | 0.85 | 0.86 | 0.87 | 0.87 | 0.88 |
|  | Ste+stop | 0.69 | 0.76 | 0.79 | 0.82 | 0.83 | 0.85 | 0.86 | 0.87 |
|  | Lem | 0.80 | 0.84 | 0.87 | 0.88 | 0.88 | 0.89 | 0.89 | 0.90 |
|  | Lem+stop | 0.75 | 0.80 | 0.84 | 0.85 | 0.87 | 0.88 | 0.88 | 0.88 |
| DVD | No-pre | 0.82 | 0.80 | 0.78 | 0.76 | 0.75 | 0.72 | 0.70 | 0.68 |
|  | Stop | 0.81 | 0.82 | 0.83 | 0.81 | 0.80 | 0.79 | 0.78 | 0.77 |
|  | Ste | 0.78 | 0.80 | 0.79 | 0.78 | 0.76 | 0.75 | 0.73 | 0.71 |
|  | Ste+stop | 0.80 | 0.82 | 0.81 | 0.81 | 0.79 | 0.78 | 0.76 | 0.74 |
|  | Lem | 0.82 | 0.81 | 0.79 | 0.77 | 0.76 | 0.74 | 0.72 | 0.71 |
|  | Lem+stop | 0.85 | 0.85 | 0.84 | 0.83 | 0.83 | 0.82 | 0.80 | 0.79 |
| WVD | No-pre | 0.64 | 0.58 | 0.56 | 0.55 | 0.54 | 0.52 | 0.50 | 0.48 |
|  | Stop | 0.70 | 0.72 | 0.71 | 0.68 | 0.67 | 0.66 | 0.62 | 0.61 |
|  | Ste | 0.66 | 0.71 | 0.67 | 0.65 | 0.63 | 0.63 | 0.59 | 0.57 |
|  | Ste+stop | 0.64 | 0.70 | 0.71 | 0.71 | 0.70 | 0.69 | 0.66 | 0.64 |
|  | Lem | 0.65 | 0.64 | 0.63 | 0.61 | 0.58 | 0.57 | 0.55 | 0.55 |
|  | Lem+stop | 0.67 | 0.70 | 0.72 | 0.73 | 0.72 | 0.71 | 0.67 | 0.68 |

**Appendix A5: Thresholds for dictionary-based techniques**

In the case of dictionary-based techniques, the decision of whether the document contained information related to politics was based on the presence of politics-related terms within a document. To address the possible variation in the length of documents coming from different platforms, we relied not on the absolute numbers but on the ratio between the number of politics-related unique terms and the overall number of unique terms per document. Table A5.1 shows the optimal ratios of unique terms from the individual dictionaries to all unique terms per document for each dictionary-based technique depending on a specific preprocessing mode. The document was to be classified as political if the ratio of unique political to non-political terms was equal to or exceeded the optimal ratio. For instance, in the case of the Di-CAP dictionary with no preprocessing mode, at least 0.48% of all unique terms present in the document had to be in the political dictionary for the document to be classified as political.

**Table A5.1**. Optimal thresholds for dictionary-based techniques for detecting politics-related information

| Preprocessing mode | Di-CAP | Di-LL | Di-CAP-LL |
| --- | --- | --- | --- |
| No-preprocessing | 0.0048 | 0.0077 | 0.0131 |
| Stopword removal | 0.0041 | 0.0093 | 0.0138 |
| Stemming | 0.0080 | 0.0056 | 0.0140 |
| Stemming+stopword removal | 0.0052 | 0.0049 | 0.0117 |
| Lemmatisation | 0.0048 | 0.0043 | 0.0089 |
| Lemmatisation+stopword removal | 0.0049 | 0.0050 | 0.0094 |

**Appendix A6: Performance metrics for politics-related information detection models**

This appendix provides a detailed overview of the performance metrics for the politics-related information detection models. Specifically, it provides information about the model performance for non-political (i.e. 0 [non-pol class]) and political (i.e. 1[pol class]) detection together with the average performance values. Three metrics are provided: (1) *precision* (pr), which shows how many of the cases identified by the model as positive were actually positive, is calculated by dividing the number of true positives by the sum of true positives and false negatives; (2) *recall* (rec), which shows how many of all the positive cases available were correctly identified by the model, is calculated by dividing the true positive rate by the sum of true positives and false positives; and (3) F1 score (F1), which is the harmonic mean of precision and recall.

The metrics are provided in Tables A6.1 to A6.6. Each table corresponds to one of the six modes of preprocessing (e.g. no preprocessing or stemming only) and contains information about the performance of the models across the following three validation datasets: (1) a *test validation* *dataset* (TVD) made of a subsample of training data (805 journalistic stories; 20% of the training sample); (2) a *diverse validation dataset* (DVD) comprising 594 short (e.g. tweets) and long content pieces (e.g. articles from German right-wing validation outlets); and (3) a *web-tracking validation dataset* (WVD) consisting of 262 documents from the corpus of web-tracking data.

**Table A6.1.** Comparison of techniques for detecting politics-related information (lowercasing/no preprocessing)

|  | TVD | | | DVD | | | WVD | | |
| --- | --- | --- | --- | --- | --- | --- | --- | --- | --- |
|  | Pr | Rec | F1 | Pr | Rec | F1 | Pr | Rec | F1 |
| *Di-CAP [av]* | 0.79 | 0.81 | 0.79 | 0.72 | 0.77 | 0.68 | 0.86 | 0.84 | 0.85 |
| 0 [non-pol] | 0.90 | 0.75 | 0.82 | 0.47 | 0.96 | 0.63 | 0.87 | 0.92 | 0.89 |
| 1 [pol] | 0.68 | 0.86 | 0.76 | 0.97 | 0.58 | 0.72 | 0.85 | 0.77 | 0.81 |
| *Di-LL [av]* | 0.75 | 0.75 | 0.71 | 0.60 | 0.62 | 0.58 | 0.75 | 0.75 | 0.75 |
| 0 [non-pol] | 0.92 | 0.59 | 0.72 | 0.38 | 0.69 | 0.49 | 0.81 | 0.81 | 0.81 |
| 1 [pol] | 0.58 | 0.92 | 0.71 | 0.60 | 0.62 | 0.58 | 0.68 | 0.68 | 0.68 |
| *Di-CAP-LL [av]* | 0.79 | 0.80 | 0.77 | 0.69 | 0.74 | 0.68 | 0.81 | 0.79 | 0.79 |
| 0 [non-pol] | 0.93 | 0.68 | 0.79 | 0.48 | 0.83 | 0.61 | 0.82 | 0.89 | 0.86 |
| 1 [pol] | 0.64 | 0.92 | 0.76 | 0.91 | 0.65 | 0.75 | 0.79 | 0.68 | 0.73 |
| *CSML [PA] [av]* | 0.91 | 0.9 | 0.91 | 0.68 | 0.66 | 0.51 | 0.81 | 0.65 | 0.65 |
| 0 [non-pol] | 0.91 | 0.95 | 0.93 | 0.36 | 0.99 | 0.53 | 0.71 | 0.98 | 0.82 |
| 1 [pol] | 0.91 | 0.85 | 0.88 | 0.99 | 0.33 | 0.49 | 0.91 | 0.32 | 0.47 |
| *CSML [BNB] [av]* | 0.9 | 0.89 | 0.89 | 0.67 | 0.61 | 0.43 | 0.83 | 0.56 | 0.51 |
| 0 [non-pol] | 0.91 | 0.93 | 0.92 | 0.33 | 1 | 0.5 | 0.66 | 1.00 | 0.79 |
| 1 [pol] | 0.88 | 0.85 | 0.86 | 1 | 0.22 | 0.36 | 1.00 | 0.12 | 0.22 |
| *CSML [MNB] [av]* | 0.9 | 0.91 | 0.9 | 0.71 | 0.75 | 0.65 | 0.83 | 0.64 | 0.63 |
| 0 [non-pol] | 0.95 | 0.9 | 0.92 | 0.45 | 0.98 | 0.61 | 0.70 | 0.99 | 0.82 |
| 1 [pol] | 0.85 | 0.92 | 0.88 | 0.98 | 0.53 | 0.69 | 0.97 | 0.29 | 0.44 |
| *CSML [LR] [av]* | 0.89 | 0.89 | 0.89 | 0.67 | 0.64 | 0.48 | 0.86 | 0.67 | 0.67 |
| 0 [non-pol] | 0.92 | 0.91 | 0.92 | 0.35 | 0.99 | 0.51 | 0.72 | 1.00 | 0.83 |
| 1 [pol] | 0.85 | 0.88 | 0.87 | 0.99 | 0.28 | 0.44 | 1.00 | 0.34 | 0.50 |
| *CSML [SGD] [av]* | 0.86 | 0.86 | 0.86 | 0.68 | 0.72 | 0.61 | 0.66 | 0.62 | 0.63 |
| 0 [non-pol] | 0.91 | 0.87 | 0.89 | 0.42 | 0.93 | 0.58 | 0.70 | 0.86 | 0.77 |
| 1 [pol] | 0.8 | 0.86 | 0.83 | 0.95 | 0.5 | 0.65 | 0.62 | 0.39 | 0.48 |
| *DL [CNN] [av]* | 0.86 | 0.78 | 0.80 | 0.65 | 0.54 | 0.30 | 0.82 | 0.53 | 0.45 |
| 0 [non-pol] | 0.80 | 0.97 | 0.88 | 0.30 | 1.00 | 0.46 | 0.64 | 1.00 | 0.78 |
| 1 [pol] | 0.92 | 0.60 | 0.73 | 1.00 | 0.08 | 0.15 | 1.00 | 0.06 | 0.12 |
| *DL [LSTM] [av]* | 0.82 | 0.83 | 0.83 | 0.65 | 0.65 | 0.53 | 0.62 | 0.56 | 0.54 |
| 0 [non-pol] | 0.89 | 0.84 | 0.86 | 0.37 | 0.93 | 0.53 | 0.66 | 0.91 | 0.76 |
| 1 [pol] | 0.76 | 0.83 | 0.79 | 0.93 | 0.37 | 0.53 | 0.58 | 0.21 | 0.31 |
| *DL [BERT] [av]* | 0.87 | 0.89 | 0.88 | 0.77 | 0.82 | 0.78 | 0.72 | 0.58 | 0.56 |
| 0 [non-pol] | 0.95 | 0.84 | 0.90 | 0.60 | 0.86 | 0.71 | 0.67 | 0.96 | 0.79 |
| 1 [pol] | 0.79 | 0.93 | 0.86 | 0.93 | 0.77 | 0.85 | 0.77 | 0.20 | 0.32 |

**Table A6.2**. Comparison of techniques for detecting politics-related information (lowercasing/no stopwords)

|  | TVD | | | DVD | | | WVD | | |
| --- | --- | --- | --- | --- | --- | --- | --- | --- | --- |
|  | Pr | Rec | F1 | Pr | Rec | F1 | Pr | Rec | F1 |
| *Di-CAP [av]* | 0.78 | 0.80 | 0.77 | 0.73 | 0.78 | 0.70 | 0.82 | 0.83 | 0.83 |
| 0 [non-pol] | 0.92 | 0.69 | 0.79 | 0.49 | 0.95 | 0.64 | 0.88 | 0.85 | 0.87 |
| 1 [pol] | 0.64 | 0.90 | 0.75 | 0.97 | 0.61 | 0.75 | 0.77 | 0.81 | 0.79 |
| *Di-LL [av]* | 0.75 | 0.75 | 0.70 | 0.58 | 0.60 | 0.57 | 0.73 | 0.72 | 0.73 |
| 0 [non-pol] | 0.93 | 0.56 | 0.70 | 0.37 | 0.64 | 0.47 | 0.79 | 0.82 | 0.80 |
| 1 [pol] | 0.56 | 0.93 | 0.70 | 0.80 | 0.57 | 0.66 | 0.67 | 0.63 | 0.65 |
| *Di-CAP-LL [av]* | 0.76 | 0.77 | 0.73 | 0.66 | 0.70 | 0.66 | 0.81 | 0.81 | 0.81 |
| 0 [non-pol] | 0.94 | 0.60 | 0.73 | 0.47 | 0.72 | 0.56 | 0.85 | 0.87 | 0.86 |
| 1 [pol] | 0.59 | 0.94 | 0.72 | 0.86 | 0.68 | 0.76 | 0.77 | 0.74 | 0.76 |
| *CSML [PA] [av]* | 0.92 | 0.91 | 0.91 | 0.68 | 0.65 | 0.5 | 0.83 | 0.64 | 0.63 |
| 0 [non-pol] | 0.92 | 0.95 | 0.93 | 0.36 | 0.99 | 0.53 | 0.70 | 0.99 | 0.82 |
| 1 [pol] | 0.91 | 0.87 | 0.89 | 0.99 | 0.31 | 0.47 | 0.97 | 0.29 | 0.44 |
| *CSML [BNB] [av]* | 0.9 | 0.89 | 0.89 | 0.67 | 0.61 | 0.43 | 0.83 | 0.59 | 0.55 |
| 0 [non-pol] | 0.91 | 0.93 | 0.92 | 0.33 | 1 | 0.5 | 0.67 | 1.00 | 0.80 |
| 1 [pol] | 0.88 | 0.85 | 0.86 | 1 | 0.22 | 0.36 | 1.00 | 0.17 | 0.30 |
| *CSML [MNB] [av]* | 0.9 | 0.91 | 0.91 | 0.71 | 0.75 | 0.65 | 0.82 | 0.64 | 0.63 |
| 0 [non-pol] | 0.95 | 0.9 | 0.93 | 0.44 | 0.98 | 0.61 | 0.70 | 0.99 | 0.82 |
| 1 [pol] | 0.85 | 0.93 | 0.89 | 0.98 | 0.53 | 0.69 | 0.93 | 0.29 | 0.44 |
| *CSML [LR] [av]* | 0.88 | 0.88 | 0.88 | 0.67 | 0.63 | 0.46 | 0.86 | 0.66 | 0.66 |
| 0 [non-pol] | 0.91 | 0.91 | 0.91 | 0.34 | 1 | 0.51 | 0.71 | 1.00 | 0.83 |
| 1 [pol] | 0.85 | 0.86 | 0.86 | 1 | 0.26 | 0.41 | 1.00 | 0.33 | 0.49 |
| *CSML [SGD] [av]* | 0.89 | 0.88 | 0.89 | 0.67 | 0.61 | 0.43 | 0.85 | 0.65 | 0.65 |
| 0 [non-pol] | 0.91 | 0.92 | 0.91 | 0.33 | 1 | 0.5 | 0.71 | 1.00 | 0.83 |
| 1 [pol] | 0.87 | 0.85 | 0.86 | 1 | 0.22 | 0.37 | 1.00 | 0.31 | 0.47 |
| *DL [CNN] [av]* | 0.89 | 0.85 | 0.86 | 0.66 | 0.58 | 0.38 | 0.80 | 0.58 | 0.54 |
| 0 [non-pol] | 0.86 | 0.96 | 0.91 | 0.32 | 1.00 | 0.48 | 0.67 | 0.99 | 0.80 |
| 1 [pol] | 0.92 | 0.74 | 0.82 | 1.00 | 0.16 | 0.27 | 0.94 | 0.16 | 0.28 |
| *DL [LSTM] [av]* | 0.91 | 0.89 | 0.90 | 0.67 | 0.63 | 0.48 | 0.78 | 0.60 | 0.58 |
| 0 [non-pol] | 0.91 | 0.95 | 0.93 | 0.35 | 0.99 | 0.52 | 0.68 | 0.98 | 0.80 |
| 1 [pol] | 0.91 | 0.84 | 0.87 | 0.98 | 0.28 | 0.44 | 0.88 | 0.22 | 0.36 |
| *DL [BERT] [av]* | 0.84 | 0.85 | 0.82 | 0.82 | 0.84 | 0.83 | 0.77 | 0.70 | 0.71 |
| 0 [non-pol] | 0.98 | 0.73 | 0.84 | 0.72 | 0.80 | 0.76 | 0.74 | 0.93 | 0.83 |
| 1 [pol] | 0.69 | 0.98 | 0.81 | 0.92 | 0.88 | 0.90 | 0.80 | 0.46 | 0.58 |

**Table A6.3**. Comparison of techniques for detecting politics-related information (lowercasing/stemming)

|  | TVD | | | DVD | | | WVD | | |
| --- | --- | --- | --- | --- | --- | --- | --- | --- | --- |
|  | Pr | Rec | F1 | Pr | Rec | F1 | Pr | Rec | F1 |
| *Di-CAP [av]* | 0.72 | 0.74 | 0.71 | 0.65 | 0.69 | 0.65 | 0.74 | 0.71 | 0.72 |
| 0 [non-pol] | 0.87 | 0.62 | 0.72 | 0.45 | 0.72 | 0.56 | 0.76 | 0.88 | 0.82 |
| 1 [pol] | 0.58 | 0.85 | 0.69 | 0.86 | 0.66 | 0.75 | 0.73 | 0.54 | 0.62 |
| *Di-LL [av]* | 0.70 | 0.71 | 0.67 | 0.58 | 0.60 | 0.55 | 0.66 | 0.67 | 0.66 |
| 0 [non-pol] | 0.87 | 0.56 | 0.68 | 0.36 | 0.68 | 0.47 | 0.76 | 0.70 | 0.73 |
| 1 [pol] | 0.54 | 0.86 | 0.67 | 0.81 | 0.52 | 0.64 | 0.56 | 0.63 | 0.59 |
| *Di-CAP-LL [av]* | 0.75 | 0.75 | 0.71 | 0.66 | 0.69 | 0.67 | 0.75 | 0.72 | 0.72 |
| 0 [non-pol] | 0.91 | 0.60 | 0.72 | 0.48 | 0.68 | 0.56 | 0.77 | 0.87 | 0.82 |
| 1 [pol] | 0.58 | 0.91 | 0.71 | 0.85 | 0.71 | 0.77 | 0.72 | 0.56 | 0.63 |
| *CSML [PA] [av]* | 0.91 | 0.9 | 0.9 | 0.68 | 0.67 | 0.53 | 0.80 | 0.65 | 0.65 |
| 0 [non-pol] | 0.92 | 0.94 | 0.93 | 0.37 | 0.99 | 0.54 | 0.71 | 0.98 | 0.82 |
| 1 [pol] | 0.89 | 0.86 | 0.88 | 0.99 | 0.36 | 0.52 | 0.89 | 0.33 | 0.48 |
| *CSML [BNB] [av]* | 0.88 | 0.88 | 0.88 | 0.67 | 0.62 | 0.44 | 0.83 | 0.58 | 0.54 |
| 0 [non-pol] | 0.91 | 0.9 | 0.91 | 0.33 | 1 | 0.5 | 0.67 | 1.00 | 0.80 |
| 1 [pol] | 0.84 | 0.85 | 0.85 | 1 | 0.23 | 0.38 | 1.00 | 0.16 | 0.28 |
| *CSML [MNB] [av]* | 0.89 | 0.9 | 0.89 | 0.69 | 0.71 | 0.6 | 0.84 | 0.6 | 0.57 |
| 0 [non-pol] | 0.94 | 0.9 | 0.92 | 0.41 | 0.96 | 0.57 | 0.68 | 1.00 | 0.81 |
| 1 [pol] | 0.84 | 0.9 | 0.87 | 0.97 | 0.47 | 0.63 | 1.00 | 0.20 | 0.34 |
| *CSML [LR] [av]* | 0.88 | 0.89 | 0.89 | 0.67 | 0.65 | 0.49 | 0.86 | 0.67 | 0.67 |
| 0 [non-pol] | 0.93 | 0.9 | 0.91 | 0.35 | 0.99 | 0.52 | 0.72 | 1.00 | 0.83 |
| 1 [pol] | 0.84 | 0.88 | 0.86 | 0.99 | 0.3 | 0.46 | 1.00 | 0.34 | 0.50 |
| *CSML [SGD] [av]* | 0.89 | 0.88 | 0.89 | 0.66 | 0.68 | 0.57 | 0.66 | 0.60 | 0.59 |
| 0 [non-pol] | 0.9 | 0.93 | 0.92 | 0.38 | 0.93 | 0.54 | 0.68 | 0.90 | 0.78 |
| 1 [pol] | 0.88 | 0.84 | 0.86 | 0.94 | 0.43 | 0.59 | 0.64 | 0.31 | 0.41 |
| *DL [CNN] [av]* | 0.58 | 0.59 | 0.58 | 0.46 | 0.47 | 0.34 | 0.53 | 0.52 | 0.51 |
| 0 [non-pol] | 0.69 | 0.67 | 0.68 | 0.26 | 0.75 | 0.39 | 0.64 | 0.81 | 0.72 |
| 1 [pol] | 0.48 | 0.50 | 0.49 | 0.65 | 0.19 | 0.29 | 0.43 | 0.23 | 0.30 |
| *DL [LSTM] [av]* | 0.87 | 0.88 | 0.88 | 0.69 | 0.68 | 0.55 | 0.78 | 0.61 | 0.59 |
| 0 [non-pol] | 0.91 | 0.90 | 0.90 | 0.38 | 0.99 | 0.55 | 0.68 | 0.98 | 0.80 |
| 1 [pol] | 0.84 | 0.85 | 0.85 | 0.99 | 0.37 | 0.54 | 0.88 | 0.23 | 0.37 |
| *DL [BERT] [av]* | 0.83 | 0.85 | 0.83 | 0.78 | 0.80 | 0.79 | 0.73 | 0.67 | 0.67 |
| 0 [non-pol] | 0.97 | 0.75 | 0.84 | 0.66 | 0.76 | 0.71 | 0.73 | 0.91 | 0.81 |
| 1 [pol] | 0.70 | 0.96 | 0.81 | 0.90 | 0.84 | 0.87 | 0.74 | 0.43 | 0.54 |

**Table A6.4**. Comparison of techniques for detecting politics-related information (lowercasing/ stemming/no stopwords)

|  | TVD | | | DVD | | | WVD | | |
| --- | --- | --- | --- | --- | --- | --- | --- | --- | --- |
|  | Pr | Rec | F1 | Pr | Rec | F1 | Pr | Rec | F1 |
| *Di-CAP [av]* | 0.72 | 0.73 | 0.70 | 0.66 | 0.70 | 0.63 | 0.76 | 0.77 | 0.77 |
| 0 [non-pol] | 0.87 | 0.62 | 0.72 | 0.43 | 0.84 | 0.57 | 0.85 | 0.78 | 0.81 |
| 1 [pol] | 0.58 | 0.85 | 0.69 | 0.90 | 0.56 | 0.69 | 0.68 | 0.77 | 0.72 |
| *Di-LL [av]* | 0.75 | 0.77 | 0.74 | 0.64 | 0.67 | 0.58 | 0.66 | 0.67 | 0.66 |
| 0 [non-pol] | 0.89 | 0.67 | 0.76 | 0.39 | 0.85 | 0.54 | 0.78 | 0.66 | 0.71 |
| 1 [pol] | 0.62 | 0.86 | 0.72 | 0.89 | 0.48 | 0.63 | 0.54 | 0.68 | 0.61 |
| *Di-CAP-LL [av]* | 0.77 | 0.78 | 0.75 | 0.68 | 0.72 | 0.66 | 0.75 | 0.74 | 0.75 |
| 0 [non-pol] | 0.90 | 0.68 | 0.78 | 0.46 | 0.84 | 0.59 | 0.80 | 0.84 | 0.82 |
| 1 [pol] | 0.63 | 0.88 | 0.73 | 0.91 | 0.61 | 0.73 | 0.71 | 0.64 | 0.67 |
| *CSML [PA] [av]* | 0.91 | 0.9 | 0.91 | 0.68 | 0.66 | 0.51 | 0.80 | 0.63 | 0.62 |
| 0 [non-pol] | 0.92 | 0.95 | 0.93 | 0.36 | 0.99 | 0.53 | 0.69 | 0.98 | 0.81 |
| 1 [pol] | 0.91 | 0.86 | 0.88 | 0.99 | 0.33 | 0.5 | 0.90 | 0.28 | 0.42 |
| *CSML [BNB] [av]* | 0.88 | 0.89 | 0.88 | 0.67 | 0.64 | 0.48 | 0.83 | 0.57 | 0.51 |
| 0 [non-pol] | 0.92 | 0.91 | 0.91 | 0.35 | 1 | 0.52 | 0.66 | 1.00 | 0.79 |
| 1 [pol] | 0.85 | 0.87 | 0.86 | 1 | 0.28 | 0.44 | 1.00 | 0.13 | 0.23 |
| *CSML [MNB] [av]* | 0.9 | 0.91 | 0.9 | 0.7 | 0.74 | 0.64 | 0.82 | 0.65 | 0.64 |
| 0 [non-pol] | 0.95 | 0.89 | 0.92 | 0.44 | 0.96 | 0.6 | 0.70 | 0.99 | 0.82 |
| 1 [pol] | 0.84 | 0.93 | 0.88 | 0.97 | 0.52 | 0.68 | 0.94 | 0.31 | 0.46 |
| *CSML [LR] [av]* | 0.89 | 0.9 | 0.89 | 0.67 | 0.64 | 0.48 | 0.84 | 0.66 | 0.66 |
| 0 [non-pol] | 0.92 | 0.92 | 0.92 | 0.35 | 1 | 0.52 | 0.71 | 0.99 | 0.83 |
| 1 [pol] | 0.87 | 0.87 | 0.87 | 1 | 0.28 | 0.43 | 0.97 | 0.33 | 0.49 |
| *CSML [SGD] [av]* | 0.88 | 0.88 | 0.88 | 0.66 | 0.68 | 0.58 | 0.64 | 0.58 | 0.57 |
| 0 [non-pol] | 0.91 | 0.91 | 0.91 | 0.39 | 0.9 | 0.55 | 0.67 | 0.89 | 0.77 |
| 1 [pol] | 0.85 | 0.85 | 0.85 | 0.93 | 0.46 | 0.62 | 0.60 | 0.28 | 0.38 |
| *DL [CNN] [av]* | 0.88 | 0.86 | 0.87 | 0.67 | 0.61 | 0.43 | 0.82 | 0.61 | 0.59 |
| 0 [non-pol] | 0.87 | 0.94 | 0.90 | 0.33 | 1.00 | 0.50 | 0.68 | 0.99 | 0.81 |
| 1 [pol] | 0.88 | 0.78 | 0.83 | 1.00 | 0.22 | 0.36 | 0.96 | 0.23 | 0.38 |
| *DL [LSTM] [av]* | 0.82 | 0.83 | 0.83 | 0.65 | 0.66 | 0.54 | 0.63 | 0.61 | 0.61 |
| 0 [non-pol] | 0.88 | 0.85 | 0.87 | 0.37 | 0.93 | 0.53 | 0.69 | 0.83 | 0.76 |
| 1 [pol] | 0.77 | 0.82 | 0.79 | 0.94 | 0.38 | 0.54 | 0.58 | 0.39 | 0.46 |
| *DL [BERT] [av]* | 0.82 | 0.83 | 0.79 | 0.80 | 0.82 | 0.81 | 0.72 | 0.71 | 0.71 |
| 0 [non-pol] | 0.99 | 0.67 | 0.80 | 0.70 | 0.76 | 0.73 | 0.77 | 0.84 | 0.80 |
| 1 [pol] | 0.65 | 0.99 | 0.79 | 0.90 | 0.87 | 0.89 | 0.68 | 0.58 | 0.63 |

**Table A6.5**. Comparison of techniques for detecting politics-related information (lowercasing/lemmatisation)

|  | TVD | | | DVD | | | WVD | | |
| --- | --- | --- | --- | --- | --- | --- | --- | --- | --- |
|  | Pr | Rec | F1 | Pr | Rec | F1 | Pr | Rec | F1 |
| *Di-CAP [av]* | 0.79 | 0.80 | 0.78 | 0.73 | 0.78 | 0.70 | 0.86 | 0.85 | 0.85 |
| 0 [non-pol] | 0.91 | 0.73 | 0.81 | 0.49 | 0.95 | 0.64 | 0.88 | 0.91 | 0.89 |
| 1 [pol] | 0.67 | 0.88 | 0.76 | 0.97 | 0.61 | 0.75 | 0.84 | 0.79 | 0.81 |
| *Di-LL [av]* | 0.70 | 0.71 | 0.69 | 0.59 | 0.60 | 0.52 | 0.70 | 0.70 | 0.70 |
| 0 [non-pol] | 0.83 | 0.64 | 0.72 | 0.34 | 0.78 | 0.48 | 0.78 | 0.77 | 0.77 |
| 1 [pol] | 0.57 | 0.79 | 0.66 | 0.83 | 0.42 | 0.56 | 0.62 | 0.63 | 0.63 |
| *Di-CAP-LL [av]* | 0.78 | 0.79 | 0.77 | 0.69 | 0.74 | 0.66 | 0.84 | 0.83 | 0.83 |
| 0 [non-pol] | 0.91 | 0.70 | 0.79 | 0.46 | 0.87 | 0.60 | 0.86 | 0.90 | 0.88 |
| 1 [pol] | 0.64 | 0.88 | 0.74 | 0.92 | 0.60 | 0.73 | 0.81 | 0.76 | 0.78 |
| *CSML [PA] [av]* | 0.92 | 0.91 | 0.91 | 0.67 | 0.66 | 0.51 | 0.78 | 0.64 | 0.63 |
| 0 [non-pol] | 0.92 | 0.95 | 0.94 | 0.36 | 0.99 | 0.53 | 0.70 | 0.97 | 0.81 |
| 1 [pol] | 0.91 | 0.87 | 0.89 | 0.99 | 0.33 | 0.5 | 0.86 | 0.31 | 0.45 |
| *CSML [BNB] [av]* | 0.9 | 0.9 | 0.9 | 0.67 | 0.62 | 0.44 | 0.83 | 0.56 | 0.51 |
| 0 [non-pol] | 0.93 | 0.92 | 0.92 | 0.33 | 1 | 0.5 | 0.66 | 1.00 | 0.79 |
| 1 [pol] | 0.87 | 0.89 | 0.88 | 1 | 0.23 | 0.38 | 1.00 | 0.12 | 0.22 |
| *CSML [MNB] [av]* | 0.9 | 0.91 | 0.91 | 0.7 | 0.74 | 0.63 | 0.82 | 0.62 | 0.60 |
| 0 [non-pol] | 0.95 | 0.9 | 0.93 | 0.43 | 0.96 | 0.6 | 0.69 | 0.99 | 0.81 |
| 1 [pol] | 0.85 | 0.92 | 0.89 | 0.97 | 0.51 | 0.67 | 0.96 | 0.24 | 0.39 |
| *CSML [LR] [av]* | 0.87 | 0.88 | 0.88 | 0.67 | 0.64 | 0.48 | 0.86 | 0.67 | 0.68 |
| 0 [non-pol] | 0.92 | 0.88 | 0.9 | 0.35 | 0.99 | 0.52 | 0.72 | 1.00 | 0.84 |
| 1 [pol] | 0.82 | 0.88 | 0.85 | 0.99 | 0.28 | 0.44 | 1.00 | 0.35 | 0.52 |
| *CSML [SGD] [av]* | 0.89 | 0.89 | 0.89 | 0.65 | 0.67 | 0.56 | 0.75 | 0.66 | 0.66 |
| 0 [non-pol] | 0.9 | 0.93 | 0.92 | 0.38 | 0.92 | 0.54 | 0.72 | 0.94 | 0.81 |
| 1 [pol] | 0.88 | 0.84 | 0.86 | 0.93 | 0.42 | 0.58 | 0.79 | 0.38 | 0.51 |
| *DL [CNN] [av]* | 0.88 | 0.85 | 0.86 | 0.66 | 0.60 | 0.41 | 0.78 | 0.60 | 0.57 |
| 0 [non-pol] | 0.86 | 0.95 | 0.90 | 0.33 | 1.00 | 0.49 | 0.68 | 0.98 | 0.80 |
| 1 [pol] | 0.90 | 0.75 | 0.82 | 1.00 | 0.19 | 0.32 | 0.88 | 0.21 | 0.34 |
| *DL [LSTM] [av]* | 0.88 | 0.89 | 0.88 | 0.67 | 0.66 | 0.53 | 0.75 | 0.60 | 0.58 |
| 0 [non-pol] | 0.92 | 0.89 | 0.91 | 0.37 | 0.97 | 0.54 | 0.68 | 0.97 | 0.80 |
| 1 [pol] | 0.84 | 0.88 | 0.86 | 0.97 | 0.36 | 0.52 | 0.82 | 0.23 | 0.37 |
| *DL [BERT] [av]* | 0.86 | 0.88 | 0.87 | 0.78 | 0.82 | 0.79 | 0.78 | 0.64 | 0.63 |
| 0 [non-pol] | 0.96 | 0.82 | 0.88 | 0.63 | 0.84 | 0.72 | 0.70 | 0.97 | 0.81 |
| 1 [pol] | 0.76 | 0.95 | 0.85 | 0.93 | 0.80 | 0.86 | 0.86 | 0.31 | 0.45 |

**Table A6.6**. Comparison of techniques for detecting politics-related information (lowercasing/ lemmatisation/no stopwords)

|  | TVD | | | DVD | | | WVD | | |
| --- | --- | --- | --- | --- | --- | --- | --- | --- | --- |
|  | Pr | Rec | F1 | Pr | Rec | F1 | Pr | Rec | F1 |
| *Di-CAP [av]* | 0.78 | 0.80 | 0.77 | 0.73 | 0.78 | 0.70 | 0.85 | 0.85 | 0.85 |
| 0 [non-pol] | 0.91 | 0.70 | 0.79 | 0.50 | 0.93 | 0.65 | 0.89 | 0.88 | 0.89 |
| 1 [pol] | 0.65 | 0.89 | 0.75 | 0.96 | 0.63 | 0.76 | 0.81 | 0.82 | 0.81 |
| *Di-LL [av]* | 0.70 | 0.71 | 0.69 | 0.58 | 0.59 | 0.51 | 0.70 | 0.70 | 0.70 |
| 0 [non-pol] | 0.84 | 0.63 | 0.72 | 0.34 | 0.76 | 0.47 | 0.78 | 0.78 | 0.78 |
| 1 [pol] | 0.57 | 0.80 | 0.67 | 0.82 | 0.42 | 0.56 | 0.63 | 0.62 | 0.63 |
| *Di-CAP-LL [av]* | 0.77 | 0.78 | 0.75 | 0.58 | 0.59 | 0.51 | 0.82 | 0.82 | 0.82 |
| 0 [non-pol] | 0.92 | 0.66 | 0.77 | 0.34 | 0.76 | 0.47 | 0.87 | 0.87 | 0.87 |
| 1 [pol] | 0.62 | 0.91 | 0.74 | 0.82 | 0.42 | 0.56 | 0.78 | 0.78 | 0.78 |
| *CSML [PA] [av]* | 0.92 | 0.91 | 0.91 | 0.67 | 0.66 | 0.51 | 0.78 | 0.63 | 0.61 |
| 0 [non-pol] | 0.92 | 0.95 | 0.93 | 0.36 | 0.99 | 0.53 | 0.69 | 0.98 | 0.81 |
| 1 [pol] | 0.92 | 0.86 | 0.89 | 0.99 | 0.32 | 0.49 | 0.87 | 0.28 | 0.42 |
| *CSML [BNB] [av]* | 0.9 | 0.9 | 0.9 | 0.67 | 0.64 | 0.47 | 0.83 | 0.56 | 0.51 |
| 0 [non-pol] | 0.93 | 0.92 | 0.92 | 0.35 | 1 | 0.51 | 0.66 | 1.00 | 0.79 |
| 1 [pol] | 0.87 | 0.89 | 0.88 | 1 | 0.27 | 0.43 | 1.00 | 0.12 | 0.22 |
| *CSML [MNB] [av]* | 0.9 | 0.91 | 0.9 | 0.72 | 0.77 | 0.67 | 0.80 | 0.63 | 0.62 |
| 0 [non-pol] | 0.95 | 0.9 | 0.92 | 0.46 | 0.98 | 0.63 | 0.70 | 0.98 | 0.82 |
| 1 [pol] | 0.85 | 0.92 | 0.88 | 0.99 | 0.56 | 0.71 | 0.90 | 0.29 | 0.43 |
| *CSML [LR] [av]* | 0.89 | 0.89 | 0.89 | 0.67 | 0.63 | 0.47 | 0.85 | 0.66 | 0.66 |
| 0 [non-pol] | 0.92 | 0.91 | 0.92 | 0.35 | 1 | 0.51 | 0.71 | 1.00 | 0.83 |
| 1 [pol] | 0.86 | 0.88 | 0.87 | 1 | 0.27 | 0.42 | 1.00 | 0.32 | 0.48 |
| *CSML [SGD] [av]* | 0.87 | 0.89 | 0.88 | 0.67 | 0.7 | 0.61 | 0.71 | 0.65 | 0.66 |
| 0 [non-pol] | 0.93 | 0.88 | 0.9 | 0.41 | 0.88 | 0.56 | 0.72 | 0.90 | 0.80 |
| 1 [pol] | 0.82 | 0.89 | 0.85 | 0.92 | 0.52 | 0.66 | 0.70 | 0.41 | 0.52 |
| *DL [CNN] [av]* | 0.88 | 0.86 | 0.86 | 0.67 | 0.61 | 0.42 | 0.81 | 0.64 | 0.64 |
| 0 [non-pol] | 0.87 | 0.94 | 0.90 | 0.33 | 1.00 | 0.50 | 0.70 | 0.98 | 0.82 |
| 1 [pol] | 0.88 | 0.77 | 0.83 | 1.00 | 0.21 | 0.35 | 0.91 | 0.31 | 0.46 |
| *DL [LSTM] [av]* | 0.89 | 0.87 | 0.88 | 0.67 | 0.67 | 0.53 | 0.68 | 0.55 | 0.51 |
| 0 [non-pol] | 0.88 | 0.95 | 0.92 | 0.37 | 0.97 | 0.54 | 0.65 | 0.96 | 0.78 |
| 1 [pol] | 0.91 | 0.79 | 0.85 | 0.97 | 0.36 | 0.53 | 0.70 | 0.14 | 0.24 |
| *DL [BERT] [av]* | 0.85 | 0.87 | 0.84 | 0.83 | 0.85 | 0.84 | 0.76 | 0.72 | 0.72 |
| 0 [non-pol] | 0.98 | 0.76 | 0.86 | 0.74 | 0.81 | 0.77 | 0.76 | 0.89 | 0.82 |
| 1 [pol] | 0.72 | 0.98 | 0.83 | 0.92 | 0.89 | 0.90 | 0.75 | 0.54 | 0.63 |
